# Langzeitüberleben bei Krebs: Definitionen, Konzepte und Gestaltungsprinzipien von Survivorship-Programmen

**DOI:** 10.1007/s00103-022-03518-x

**Published:** 2022-03-17

**Authors:** Corinna Bergelt, Carsten Bokemeyer, Inken Hilgendorf, Thorsten Langer, Oliver Rick, Ulf Seifart, Uwe Koch-Gromus

**Affiliations:** 1 Institut für Medizinische Psychologie, Universitätsmedizin Greifswald, Walther-Rathenau-Str. 48, 17475 Greifswald, Deutschland; 2 II. Medizinische Klinik und Poliklinik, Universitätsklinikum Hamburg-Eppendorf, Universitäres Cancer Center, Hamburg, Deutschland; 3 Klinik für Innere Medizin II, Abteilung für Hämatologie und Internistische Onkologie, Universitätsklinikum Jena, Jena, Deutschland; 4 Klinik für Kinder- und Jugendmedizin, Universitätsklinikum Schleswig-Holstein, Lübeck, Deutschland; 5 Klinik Reinhardshöhe, Bad Wildungen, Deutschland; 6 Klinik Sonnenblick Lehrkrankenhaus Universitätsklinik Marburg, Marburg, Deutschland; 7 Institut und Poliklinik für Medizinische Psychologie, Universitätsklinikum Hamburg-Eppendorf, Hamburg, Deutschland

**Keywords:** Krebs-Langzeitüberleben, Langzeitfolgen, Spätfolgen, Biopsychosoziale Versorgungsbedarfe, Versorgungsmodelle, Cancer survivorship, Long-term sequelae, Late effects, Biopsychosocial care needs, Healthcare models

## Abstract

Aufgrund von diagnostischen und therapeutischen Fortschritten in der Hämatologie und Onkologie und entsprechend steigenden Überlebensaussichten ist ein stetiger Zuwachs der Gruppe von Langzeitüberlebenden mit und nach Krebs (Cancer Survivor) in Deutschland zu verzeichnen. Obwohl das bereits vorhandene deutsche Gesundheitswesen vielfältige Versorgungsangebote vorhält, die auch für Langzeitüberlebende verfügbar sind, ist die Versorgungssituation dieser Gruppe nicht zufriedenstellend. So bedarf es zum einen der Entwicklung von Orientierungshilfen für Langzeitüberlebende, zum anderen sollten neue und innovative Versorgungsprogramme für Überlebende (Survivorship-Programme) entwickelt werden. Der Beitrag gibt einen Überblick über die Problematik, definiert relevante Begrifflichkeiten und formuliert Kernaspekte für die Ausgestaltung von Survivorship-Programmen für Langzeitüberlebende nach Krebserkrankung.

## Abstract (en)

Due to improvements in diagnostics and treatment options in hematology and oncology, and thus increasing chances of survival, the number of long-term survivors living with and after a cancer disease in Germany is constantly rising. Although the existing German healthcare system provides multifaceted healthcare offers that are available to long-term survivors, the healthcare situation of this population is not satisfactory. Thus, orientation guides for long-term survivors as well as new and innovative survivorship programs should be developed. This paper provides an overview of the complex of problems, defines relevant concepts, and devises central topics for the development of survivorship programs for long-term cancer survivors.

## Hintergrund

Aufgrund von diagnostischen und therapeutischen Fortschritten in der Hämatologie und Onkologie und entsprechend steigenden Überlebensaussichten ist ein stetiger Zuwachs der Gruppe von Langzeitüberlebenden mit und nach Krebs (Cancer Survivors) in Deutschland zu verzeichnen. Dieser erfreulichen Entwicklung stehen jedoch häufig auch unerwünschte, individuell unterschiedlich ausgeprägte medizinische, psychische und soziale Folgen und Belastungen sowie Einschränkungen der Lebensqualität bei Betroffenen (und ihren Angehörigen) gegenüber, z. B. durch Langzeitnebenwirkungen der Therapie oder Auswirkungen der Erkrankung auf die Lebens- und Berufsperspektive. Derartige unerwünschte Nebenwirkungen gab es auch schon früher, sie standen aber gesellschaftspolitisch und medizinisch zu wenig im Fokus.

Vor diesem Hintergrund wurde im Rahmen des Nationalen Krebsplans (NKP) vom Bundesministerium für Gesundheit (BMG) eine Expert:innen-Arbeitsgruppe „Langzeitüberleben nach Krebs (AG LONKO)“ eingerichtet, um Wissenslücken hinsichtlich der Versorgungssituation von Krebslangzeitüberlebenden zu identifizieren und zu schließen und um perspektivisch bedarfsgerechte und strukturell schlüssige Versorgungskonzepte entwickeln zu können.

Für die Umsetzung dieser Aufgabe wurde eine Unterarbeitsgruppe „Bedarfsgerechte Versorgungsmodelle“^1^ eingerichtet, die inzwischen ein Empfehlungspapier formuliert hat, das in der AG LONKO konsentiert und vom NKP verabschiedet wurde [1]. Der nachfolgende Beitrag greift zentrale Teile dieses Empfehlungspapiers auf, in denen definitorische und konzeptuelle Aspekte von Langzeitüberleben und Versorgungsprogrammen für Überlebende (Survivorship-Programmen) behandelt werden.

## Langzeitüberleben nach Krebs: Verständnis und Begrifflichkeiten

Die Fortschritte in der Behandlung von Krebserkrankungen führen dazu, dass ein immer größer werdender Anteil der Patient:innen die Krankheit überlebt und im Gesundheitswesen immer mehr von einer Krebserkrankung Betroffene mit Krankheits- und Behandlungsfolgen sowie Spät- und Langzeitfolgen der Krebserkrankung und -behandlung versorgt werden müssen. Die Behandlungsmöglichkeiten wie Operation, Chemotherapie, Immuntherapie, Strahlentherapie, Zelltherapie und Hormontherapie sind zum Teil mit erheblichen Nebenwirkungen und dem Risiko von Langzeit- und Spätfolgen verbunden. Während *Langzeitfolgen* gemäß ihrer Definition [2, 3] während der Akuttherapie beginnen und jahrelang andauern können, treten *Spätfolgen* erst Monate oder auch noch viele Jahre nach Ende der Krebstherapie auf. Häufige Langzeitnebenwirkungen und Spätfolgen von Krebstherapien sind in dem Empfehlungspapier der Unterarbeitsgruppe „Datenerhebung und Datenanalyse“ der AG LONKO enthalten [4]:

- kardiovaskuläre Erkrankungen (Erkrankungen des Herzens und des Gefäßsystems),
- Sekundärmalignome (Zweittumoren),
- Polyneuropathien,
- Infertilität,
- Erschöpfung/Fatigue,
- Depressionen,
- Rezidiv- und Progredienzängste,
- wachsende berufliche Probleme,
- finanzielle und soziale Probleme.

Es hat sich etabliert, die Überlebenden der Erkrankung mit dem englischen Begriff „Survivor“ zu bezeichnen und bei Krebsüberleben von „Cancer Survivorship“ zu sprechen. Der Zeitpunkt des Beginns des Langzeitüberlebens wird in der internationalen Literatur unterschiedlich definiert. Eine weithin akzeptierte Einteilung verschiedener Stadien des Überlebens unterscheidet das frühe (akute) vom mittleren und vom Langzeitüberleben (ab 5 Jahre nach Abschluss der Akutbehandlung; [5]). Epidemiologische Schätzungen gehen davon aus, dass mindestens 4,5 Mio. Menschen in Deutschland mit oder nach einer Krebserkrankung leben und dass etwa 2 Drittel dieser Cancer Survivor als Langzeitüberlebende gelten können [6].

Da die Cancer Survivor eine Vielzahl erkrankungs- und behandlungsbedingter Langzeit- und Spätfolgen im körperlichen, funktionellen, psychischen und sozialen Bereich erleben können [7, 8], haben sie Bedarf an Versorgungsangeboten, die zum einen die verschiedenen vorhandenen Langzeit- und Spätfolgen und Problembereiche ganzheitlich adressieren und zum anderen die Wahrscheinlichkeit der Entstehung weiterer Langzeit- und Spätfolgen präventiv reduzieren. Unter Expert:innen besteht ein hoher Konsens darüber, dass das deutsche Versorgungssystem für Langzeitüberlebende bislang keine adäquat strukturierten und ganzheitlichen Versorgungsangebote systematisch vorhält und daher deutlicher Handlungsbedarf besteht. Vor allem international gibt es inzwischen umfangreiche Forschungsergebnisse zum Thema Langzeitüberleben. In einzelnen Teilbereichen liegen gut fundierte Übersichtsarbeiten vor, beispielsweise im Hinblick auf bestimmte Indikationsbereiche [9] oder Gruppen von Betroffenen [10], in anderen – ebenfalls sehr wichtigen – Bereichen ist die bisherige Forschung noch defizitär. Darüber hinaus stammen die meisten der vorliegenden Publikationen, insbesondere in Bezug auf Versorgungspläne und -modelle, aus dem angloamerikanischen Raum [11–14] und es ist offen, inwieweit eine Übertragbarkeit auf das deutsche Gesundheitssystem gegeben ist.

## Versorgung von Langzeitüberlebenden: Survivorship-Programme

Die übergeordnete Zielsetzung von Survivorship-Programmen ist es, Patient:innen nach Ende der akutmedizinischen Behandlung im Übergang von der Phase der Erkrankung und Behandlung zur Phase des Langzeitüberlebens zu begleiten und sie hinsichtlich des jeweiligen individuellen Beratungs- und Behandlungsbedarfes angemessen zu versorgen, um die mittel- und langfristigen Auswirkungen von Erkrankung und Behandlung soweit möglich zu verringern. Die Notwendigkeit einer solchen Begleitung und der Gestaltung des Übergangs wurde eindrücklich vom Institute of Medicine in der Schrift „From cancer patient to cancer survivor: lost in transition“ („Vom Krebspatienten zum Krebsüberlebenden: Verloren im Übergang“) formuliert [15].

Die übergeordneten Zielsetzungen solcher Programme sind [5]:

- Erkennen und Therapie medizinischer und psychosozialer Langzeit- und Spätfolgen,
- Angebot verschiedener Interventionen, um die Auswirkungen der Krebstherapie und -erkrankung in medizinischer und psychosozialer Hinsicht zu mindern,
- verbesserte Koordination zwischen Spezialist:innen und nachsorgenden Ärzt:innen,
- Prävention von Tumorrezidiven, Zweittumoren und anderen Spätkomplikationen.

Im deutschen Gesundheitswesen sind grundsätzlich zahlreiche Versorgungsangebote für Langzeitüberlebende nach einer Krebserkrankung vorhanden, die die genannten Zielsetzungen jeweils separat in Teilen adressieren. Neben der medizinischen Nachsorge gibt es vielfältige Angebote wie psychosoziale Krebsberatungsstellen, Rehabilitationsmaßnahmen, psychoonkologische Versorgung, therapeutische Angebote wie Physio- oder Ergotherapie etc., die – über die Versorgungssektoren verteilt – in unterschiedlichen Settings und unter verschiedenen Rahmenbedingungen nebeneinander verfügbar sind. Darüber hinaus spielen in der Versorgung von Langzeitüberlebenden auch selbsthilfebezogene Organisationen sowie digitale Informations- und Beratungsangebote (z. B. Krebsinformationsdienst des Deutschen Krebsforschungszentrums [DKFZ] [16], Junges Krebsportal [17]) eine wichtige Rolle. Diese Informations- und Beratungsangebote werden zunehmend und unvermeidlich durch wissenschaftlich ungesicherte Informationen im Internet und in den sozialen Medien überlagert.

Die Selbsthilfe ist für viele Langzeitüberlebende schon seit Langem eine bewährte Instanz in Bezug auf eine fachlich begründete Begleitung und Beratung von Betroffenen durch Betroffene – sowohl im Hinblick auf persönliche Erfahrungen im Umgang mit Langzeit- und Spätfolgen als auch auf sozialrechtliche Ansprüche sowie Möglichkeiten der Unterstützung bei der Bewältigung der psychischen Krankheitsfolgen.

Trotz bereits zahlreicher grundsätzlich für die Betreuung der Langzeitüberlebenden vorhandener Versorgungsangebote ist die Entwicklung von spezifischen ganzheitlichen Survivorship-Programmen aus verschiedenen Gründen notwendig:

Zum einen findet über diese vielfältigen verschiedenen Angebote hinweg keine systematische Bedarfserfassung der Situation und Bedürfnisse der Langzeitüberlebenden statt. Es fehlt dementsprechend eine Instanz, die einen systematischen Überblick über die Situation und die Bedarfe und Bedürfnisse der Langzeitüberlebenden hat und die eine bedarfsgerechte Versorgung steuern und eine gezielte Inanspruchnahme spezifischer Angebote initiieren könnte.

Zum anderen werden die vorhandenen Angebote für die Betroffenen weder strukturiert noch in irgendeiner Form systematisiert. Es ist also von einzelnen Behandelnden oder den Betroffenen selbst abhängig, ob spezifische Versorgungsangebote, wie beispielsweise rehabilitative Maßnahmen, oder spezifische therapeutische Interventionen zum einen bekannt sind und zum anderen in Anspruch genommen werden. Im Hinblick auf die Inanspruchnahme ist es für die Betroffenen eine zusätzliche Hürde, dass die Angebote von ganz unterschiedlichen Personen und Institutionen durchgeführt werden, über verschiedene Versorgungssektoren verteilt sind und jeweils unterschiedliche Zugangsbedingungen vorhanden sind. Dies bedingt, dass es unter Umständen vor allem von vorhandenen oder erworbenen spezifischen Kenntnissen und/oder der Eigeninitiative der Betroffenen abhängt, ob eine bestimmte Behandlung oder Therapiemaßnahme in Anspruch genommen werden kann. Vor dem Hintergrund der Belastungen der Betroffenen und ihrer insgesamt vulnerablen Situation kann dies nicht als zufriedenstellende Versorgungssituation angesehen werden.

Darüber hinaus wird die Inanspruchnahme von Behandlungsangeboten dadurch erschwert, dass die diversen Angebote nicht gleichermaßen flächendeckend vorhanden und erreichbar sind. So ist es beispielsweise in einem Ballungsraum sehr viel einfacher, Zugang zu spezifischen Angeboten, wie etwa psychoonkologischer Versorgung, zu erhalten und diese verkehrstechnisch zu erreichen als in ländlichen Gebieten.

Vor diesem Hintergrund ist trotz der umfangreichen Versorgungsangebote davon auszugehen, dass eine individuell passende bedarfsgerechte Versorgung der Betroffenen derzeit nicht systematisch sichergestellt ist. Unter Expert:innen sowie denjenigen, die in der medizinischen und psychosozialen Versorgung von Langzeitüberlebenden tätig sind, herrscht daher Konsens darüber, dass die Entwicklung von ganzheitlichen Survivorship-Programmen dringend erforderlich ist. Diese Programme für Langzeitüberlebende sollten bereits während der Akutphase der Behandlung, auch im Sinne von prähabilitativen Maßnahmen^2^ [18, 19] mitgedacht und angestoßen werden, auch wenn die Hauptaufgabe der Survivorship-Programme in der Identifikation und Versorgung von Spät- und Langzeitfolgen der Krebserkrankung besteht, ohne dass hier eine zeitliche Begrenzung festgelegt werden kann.

Die Programme müssen zum einen die Anforderung erfüllen, eine Systematisierung sowohl im Hinblick auf das Assessment von Spät- und Langzeitfolgen als auch auf eine Systematisierung und Strukturierung von Versorgungsangeboten vorzunehmen. Zum anderen müssen die Programme eine ganzheitliche Versorgung somatischer, funktioneller, psychischer und sozialer Spät- und Langzeitfolgen ermöglichen, die auf die individuelle Situation der Betroffenen zugeschnitten ist und den Bedarfen und Bedürfnissen der einzelnen Betroffenen gerecht wird. Hierzu bedarf es also klarer Registerstudien, die nach einheitlichen Kriterien medizinische und psychosoziale Aspekte erfassen und eine Auswertung großer Kohorten erlauben. Darüber hinaus müssen die Programme auch erlauben, die Effektivität eingesetzter Interventionen nach definierten Kriterien auszuwerten.

## Aktuelle Modellprojekte zur Versorgung von Langzeitüberlebenden

An den onkologischen Spitzenzentren sowie durch die Förderung von Modellvorhaben, beispielsweise im Rahmen des beim Gemeinsamen Bundesausschuss (G-BA) angesiedelten Innovationsfonds, gibt es inzwischen einzelne erste Modellprogramme, in denen eine Bedarfserfassung, Strukturierung von Angeboten und bedarfsorientierte Versorgung mit spezifischen präventiv orientierten Behandlungsbausteinen erfolgen. Zum Anforderungsprofil onkologischer Spitzenzentren gehört inzwischen auch die Einrichtung eines Angebotes für Langzeitüberlebende nach Krebs.

Seit ein paar Jahren gibt es in einzelnen Teilbereichen konzeptionell sehr gut fundierte Modellvorhaben, in denen neue interdisziplinäre Survivorship-Programme erprobt werden. Beispielhaft ist hier das am Universitären Cancer Center Hamburg entwickelte Programm CARE for CAYA^3^ [20, 21] zu nennen, das im Rahmen eines Projekts entstanden ist, welches durch den beim G‑BA angesiedelten Innovationsfonds gefördert wurde. Inzwischen wird das Programm deutschlandweit in 14 regionalen CAYA-Zentren umgesetzt und evaluiert.

## Diskussion

Aus den vorangegangenen Darstellungen geht hervor, dass in Deutschland ein breit ausgebautes onkologisches Versorgungssystem existiert, das auch wichtige Bedarfe von Langzeitüberlebenden adressiert. Nichtsdestotrotz besteht für die Versorgung von Langzeitüberlebenden ein erheblicher Entwicklungsbedarf: Es fehlt an Orientierung über bereits vorhandene Behandlungsmöglichkeiten, an einer soliden, repräsentativen Datengrundlage für (strukturierte) spezifische Interventionsprogramme und an strukturierten Survivorship-Programmen, die individuelle Risikoaspekte für Langzeit- und Spätfolgen der Langzeitüberlebenden berücksichtigen. Insofern ist es zu begrüßen, dass das BMG, wie eingangs erwähnt, im Rahmen des Nationalen Krebsplans dem Thema Survivorship Priorität einräumt und auf der Basis des vorgelegten Empfehlungspapiers eine erste Förderbekanntmachung zur Bestandsaufnahme der aktuellen Versorgungssituation veröffentlicht hat [22].

Neben einer sorgfältigen Analyse der aktuellen Versorgungssituation und Bestandsaufnahme von Interventionen für Langzeitüberlebende besteht jedoch auch ein Bedarf an der Entwicklung neuer innovativer Survivorship-Programme in Deutschland. Für die konzeptionelle Gestaltung dieser Survivorship-Programme sind nach dem Verständnis der Autor:innen die folgenden wichtigen Prinzipien hervorzuheben:

Grundanliegen eines Survivorship-Programms müssen die *frühe Identifizierung von Langzeit- und Spätfolgen* der Erkrankung und Therapie sowie das Vorhalten spezifischer Angebote für die Behandlung und das Management von Spät- und Langzeitfolgen (s. oben) sein. Darüber hinaus sollen die Ansätze *präventiv orientiert* sein, um möglichen Spät- und Langzeitfolgen vorzubeugen. Insbesondere angesichts eines breiten Einsatzes von hocheffektiven neuen Methoden in der Krebstherapie, wie z. B. Immuntherapien, zellulären Therapien und der Langzeitbehandlung mit Tyrosinkinaseinhibitoren, ist auch die Erfassung bisher unbekannter medizinischer Spätfolgen von großer Bedeutung für zukünftige Survivorship-Programme.

Die Programme müssen auch *Veränderungen in der sozialen und beruflichen Situation der Betroffenen* berücksichtigen. Besondere Aufmerksamkeit sollte dabei dem Risiko der sozialen Ungleichheit in der Versorgung gewidmet werden. Von zentraler Bedeutung sind die nach der Erkrankung gegebene berufliche Situation und die Fortsetzung der Erwerbstätigkeit oder Ausbildung, die durch Wiedereingliederungsmaßnahmen und andere Leistungen zur Teilhabe am Arbeitsleben unterstützt werden kann. Bedeutsame Einflussfaktoren sind hier die *persönliche Motivation* der Betroffenen und die *objektiven und subjektiven Bedingungen am Arbeitsplatz*.

Die Programme sollten darüber hinaus auch die *finanziellen Auswirkungen* der Krebserkrankung für die Betroffenen reflektieren. Angesichts der Bedeutung von Erwerbsarbeit für den finanziellen und sozialen Status in unserer Gesellschaft stellt die Diagnose Krebs ein relevantes Problem für die Betroffenen dar. Eine Berentung oder der krankheitsbedingte Verlust des Arbeitsplatzes haben erhebliche finanzielle Konsequenzen für die Betroffenen [23, 24].

Bei der Entwicklung und Ausgestaltung von Programmen zur Versorgung von Langzeitüberlebenden muss beachtet werden, dass unterschiedliche und zum Teil sehr *spezifische Bedarfe und Bedürfnisse verschiedener Gruppen von Betroffenen* hinsichtlich ihres *Lebensalters* und der jeweiligen *Krankheitsphase* berücksichtigt werden müssen. Bei Betroffenen im Kindes‑, Jugend- und jungen Erwachsenenalter müssen andere Schwerpunkte gesetzt werden (z. B. Autonomieentwicklung, Fertilitätserhalt, Schule und Ausbildung; [25]) als bei denjenigen im mittleren Lebensalter (z. B. Erwerbstätigkeit) und im höheren Lebensalter (z. B. Komorbiditäten und altersbedingte Einschränkungen). Darüber hinaus ist zu beachten, dass sich die Belastungen und Bedarfe der einzelnen Betroffenen im Verlauf von Behandlung, Nachsorge und Langzeitüberleben verändern.

Auch *Angehörige und enge Bezugspersonen* sollten in die Survivorship-Programme einbezogen werden, da Lebenspartner:innen, Kinder und bei jüngeren Patient:innen auch deren Eltern und Geschwisterkinder von der Erkrankung in vielerlei Hinsicht mitbetroffen sein können. Zu nennen sind hier unter anderem psychische Belastungen basierend auf der Sorge um das erkrankte Familienmitglied sowie soziale Auswirkungen durch Wegfall oder Einschränkungen von Berufstätigkeit und damit verbundene Einkommenseinbußen. Darüber hinaus übernehmen Angehörige und enge Bezugspersonen häufig pflegerische und unterstützende Tätigkeiten während der Erkrankung und im weiteren Verlauf, wenn es um das Management der Krankheitsfolgen geht.

Die Entwicklung von Survivorship-Programmen sollte *auf den bereits vorhandenen Versorgungsstrukturen aufbauen* und bestehende und sehr gut etablierte Angebote, wie beispielsweise Krebsberatungsstellen, Rehabilitationskliniken, Selbsthilfeorganisationen, und auch die medizinische Nachsorge im ambulanten Sektor im Sinne der Langzeitüberlebenden besser vernetzen. Allerdings fehlt es in diesen Bereichen bislang an koordinierenden Instanzen, um diese Angebote sinnvoll miteinander zu verbinden.

## Fazit

Langzeitüberlebende nach einer Krebserkrankung können von vielfältigen Spät- und Langzeitfolgen betroffen sein. Obwohl das deutsche Gesundheitssystem vielfältige Versorgungsangebote vorhält, fehlt es bislang an ganzheitlichen Survivorship-Programmen, in denen die besondere Situation dieser Population adäquat adressiert wird. Es ist zu begrüßen, dass diese Thematik in ersten Modellprojekten aufgegriffen und im Nationalen Krebsplan verankert wurde. Dies liefert die Voraussetzungen für die Weiterentwicklung der Versorgung für Langzeitüberlebende und für die Entwicklung und Erprobung neuer Versorgungsmodelle.

## Footnotes

1 Die Mitglieder der Unterarbeitsgruppe sind identisch mit den Autor:innen dieses Beitrages. Die Erstautorin und der Letztautor fungierten als Sprecherin und Sprecher der Unterarbeitsgruppe.

2 Prähabilitation: Durchführung multidisziplinärer Interventionen vor einer Operation oder Therapie, die darauf abzielen, Nebenwirkungen und Folgen der Operation oder Therapie zu vermindern.

3 CARE for CAYA: Comprehensive Assessments and Related Interventions to Enhance Long-term Outcome in Children, Adolescents and Young Adults.
